# Multivalent Fusion DNA Vaccine against* Brucella abortus*

**DOI:** 10.1155/2017/6535479

**Published:** 2017-09-07

**Authors:** Leonardo Gómez, Javiera Llanos, Emilia Escalona, Darwin Sáez, Francisco Álvarez, Raúl Molina, Manuel Flores, Angel Oñate

**Affiliations:** Laboratory of Molecular Immunology, Department of Microbiology, Faculty of Biological Sciences, University of Concepción, Concepción, Chile

## Abstract

As an alternative brucellosis prevention method, we evaluated the immunogenicity induced by new multivalent DNA vaccines in BALB/c mice. We constructed the vaccines by fusion of BAB1_0273 and/or BAB1_0278 open reading frames (ORFs) from genomic island 3 (GI-3) and the* Brucella abortus* 2308* sodC* gene with a link based on prolines and alanines (pV273-*sod*, pV278-*sod,* and pV273-278-*sod*, resp.). Results show that immunization with all tested multivalent DNA vaccines induced a specific humoral and cellular immune response. These novel multivalent vaccines significantly increased the production of IgM, IgG, and IgG2a antibodies as well as IFN-*γ* levels and the lymphoproliferative response of splenocytes. Although immunization with these multivalent vaccines induced a typical T-helper 1- (Th1-) dominated immune response, such immunogenicity conferred low protection levels in mice challenged with the* B. abortus* 2308 pathogenic strain. Our results demonstrated that the expression of BAB1_0273 and/or BABl_0278 antigens conjugated to SOD protein can polarize mice immunity to a Th1-type phenotype, conferring low levels of protection.

## 1. Introduction

*Brucella* spp. cause brucellosis, a globally distributed zoonosis infecting more than half a million people worldwide every year [1]. This genus is constituted by Gram-negative, coccobacillary, microaerophilic, non-spore-forming, immotile, intracellular facultative, and slow-growing bacteria [2]. Such pathogens are found in a wide range of mammalian hosts, such as* B. abortus* (bovine),* B. melitensis* (goats), and* B. suis* (pigs) which are the most pathogenic species within the genus for humans [2, 3]. Infection with these bacteria usually occurs through ingestion of contaminated food or through contact with infected animals [4]. In animals, brucellosis produces abortions in females and infertility in males, whereas in humans it is manifested by an acute phase of undulating fever and a chronic phase associated with arthritis, orchitis, hepatitis, encephalomyelitis, and endocarditis [5, 6].

*B. abortus* is a pathogen highly adapted to the intracellular environment. Inside cells, there exist adverse conditions including acidic pH, proteases, and reactive oxygen species (ROS) and reactive nitrogen species (RNS) [7]. The pathogen survives this hostile microenvironment by expressing Cu-Zn superoxide dismutase (SOD) enzyme which detoxifies the superoxide radical (O_2_^−^) transforming it into oxygen (O_2_) and hydrogen peroxide (H_2_O_2_) [8]. In addition, its traffic into the cells is carried out in a* Brucella* Containing Vacuole (BCV), which interacts with early and lysosomal endosomes that acidify the BCV, promoting the expression of several virulence factors, such as the VirB type IV secretion system (SST4) [3]. These factors allow it to avoid the phagolysosomal proteases and redirect its intracellular traffic to the endoplasmic reticulum, where it creates a replicative niche [9]. Among the molecules secreted by SST4 is the Btp1 protein, which inhibits dendritic cell maturation [10]. This protein is encoded in* B. abortus* BAB1_0279 open reading frame (ORF) of genomic island 3 (GI-3). This GI in* B. abortus* 2308 includes the ORF BAB1_0250 to BAB1_0279 and contains 25 genes, many of which have unknown function, and several pseudogenes [11].

Current vaccines used to prevent brucellosis depend on live attenuated* B. abortus* RB51 and S19 strains [12]. These vaccines have been effective in controlling the disease; however, they may cause abortions in immunized animals and are pathogenic for humans [13]. An alternative for the development of effective and safe vaccines can be achieved by the immunization with DNA, a vaccinal strategy inducing T-helper type 1 (Th1) immune responses associated with IFN-*γ* producing CD4+ T and cytotoxic CD8+ T cells and IgG2a antibody produced by B cells, required to clear* B. abortus* infection [14]. In addition, DNA vaccines allow expression of individual antigens, fused multiple antigens, or immunodominant epitopes from each antigen to enhance vaccine immunogenicity [15–17]. Among the antigens used for the development of DNA vaccines against brucellosis are BAB1_0278 and BAB1_0273 ORFs. BAB1_0278 encodes a hypothetical GrcA protein involved in the cell cycle, whose deletion affects the efficiency of bacterial growth within phagocytes and its virulence [18], and a DNA vaccine codifying this ORF showed to efficiently induce an immune response and protection [19, 20]. On the other hand, BAB1_0273 is a possible DNA-binding protein, exhibiting immunodominant epitopes used to design and to evaluate a multiple epitope DNA vaccine, which showed to be immunogenic and to confer protection in the murine model [21]. Furthermore, it has been shown that peptides of the Cu-Zn superoxide dismutase protein (Cu/Zn SOD) are immunodominant and a DNA vaccine encoding the* sodC* gene (Cu-Zn SOD) provides protection [17, 22].

Therefore, due to the immunogenic characteristics reported for the BAB1_0273, BAB1_0278, and the* sodC* genes, we designed three multivalent DNA vaccines based on the BAB1_0273 and/or BAB1_0278 ORFs fused with the* sodC* gene from* B. abortus*, with the aim of evaluating their efficiency to induce an effective immune response against brucellosis.

## 2. Materials and Methods

### 2.1. Animals

Eight-week-old BALB/c female mice were provided by the Instituto de Salud Publica, Santiago, Chile. Animal maintenance was carried out in the Molecular Immunology Laboratory (Department of Microbiology, Faculty of Biological Sciences, University of Concepción), where they were fed with water and food “ad libitum.” Management of these animals was carried out in accordance with the regulations of the Bioethics and Safety Committee of the Faculty of Biological Sciences, University of Concepción regulations. All efforts were made to minimize animal suffering.

### 2.2. Design and Construction of Multivalent DNA Vaccines

Vaccines were constructed using BAB1_0273 and BAB1_0278 ORFs and the* sodC* gene from* B. abortus* 2308, according to nucleotide sequences available from GenBank (WP_002965521.1, WP_002965527.1, and WP_002972093.1, resp.). We used three different gene combinations: BAB1_0273-*sod*, BAB1_0278-*sod,* and BAB1_0273-278-*sod*. Genes were fused using a rigid linker based on a proline and alanine sequence (PAPAP) [23]. The chimeric multivalent genes were chemically synthesized by GenScript, Inc. (Piscataway, NJ, USA), with codon optimization for mouse and* Escherichia coli*. These sequences were cloned in the* Pst*I and* Bam*HI restriction sites into the pVAX1 vector (Thermo Fisher Scientific Inc., MA, USA) and linked with the T4 ligase enzyme (New England Biolabs, Inc., MA, USA). Resulting constructs were named pV273-*sod*, pV278-*sod,* and pV273-278-*sod*, and their construction was successfully performed and visualized in 1% agarose gels. DNA required for all immunizations were obtained by electroporation of the* E. coli* DH5*α* strain with each of these vectors and cultured in Terrific Broth (TB) supplemented with 50 *μ*g/ml kanamycin during 18 h at 37°C. Subsequently, the vectors were purified using the alkaline lysis procedure [24]. The final concentrations of the recombinant DNAs (pV273-*sod*, pV278-*sod,* and pV273-278-*sod*) were quantified using the Infinite® 200 PRO NanoQuant kit (Tecan Group Ltd., Switzerland) and adjusted to the concentrations required for each immunization.

### 2.3. Expression and Purification of Recombinant Proteins

Multivalent recombinant proteins (MVRPs) encoded by BAB1_0273-*sod*, BAB1_0278-*sod,* and BAB1_0273-278-*sod* genes were purified cloning these sequences in the prokaryote pQe80L (Addgene, MA, USA) expression vector: pQ273s, pQ278s, and pQ273-278s. Later,* E. coli* BL21 (DE3) strain was transformed with these vectors to express the recombinant proteins, inducing their expression with 0.2 mM isopropyl thiogalactopyranoside (IPTG) for 4 h at 25°C. Subsequently, bacteria were resuspended in phosphate buffered saline (PBS) with 0.2 mM phenylmethylsulfonyl fluoride (PMSF) and sonicated for 10 min (70% amplitude with 30 second pulses) on ice. Proteins were detected in the soluble phase and purified by histidine affinity chromatography using HisTrap FF crude columns (GE Healthcare, UK). Recombinant protein purification was visualized by 12% sodium dodecyl sulfate polyacrylamide gel electrophoresis (SDS-PAGE) and confirmed with a western blot using 6x anti-His antibodies (Abcam, Cambridge, UK). These recombinant proteins will be hereon named R273S, R278S, and R273-278S, respectively. Proteins were quantified using the Pierce™ BCA Protein Assay kit (Thermo Fisher Scientific Inc., MA, USA) using Bovine Serum Albumin (BSA) as the standard.

### 2.4. Immunizations

Mice were randomly separated into negative control (PBS and pVAX) and experimental (pV273-sod, pV278-sod, and pV273-278-sod) groups with five animals per group. In the case of immunization with a DNA-type vaccine, each animal received a 100 *μ*g dose of recombinant DNA in 100 *μ*l of PBS, divided into two injections of 50 *μ*l in each posterior tibialis muscle. The PBS negative control received 100 *μ*l of PBS, injected as described above for the experimental group. All animals received three immunizations at 15-day intervals [25].

### 2.5. Production of Antibodies

Antibody production was quantified from the peripheral blood of mice obtained at 0, 15, 30, and 45 days after immunization using the indirect ELISA method [25]. For this, 96-well plates (Nunc Maxisorp, Thermo Fisher Scientific, MA, USA) were coated with 5 *μ*g of recombinant proteins per milliliter of carbonate-bicarbonate buffer (50 mM, pH 9.6) and incubated for 16 h at 4°C in a humidity chamber. Plates were washed with TPBS buffer (PBS plus 0.05% Tween 20) and blocked with 0.8% gelatin for 1 h at 37°C in order to avoid nonspecific binding. To each well, 100 *μ*l of serum was added in serial dilutions using TPBS plus 0.2% gelatin, starting at 1 : 200 dilutions. Samples were incubated for 3 h at 37°C. After this time, rabbit anti-mouse IgM, IgG, IgG1, or IgG2a secondary antibodies conjugated with horseradish peroxidase (Serotec, Oxford, UK) diluted 1 : 1000 were added and incubated for 45 min at 37°C. The reaction was revealed with 100 *μ*l SigmaFast (Sigma-Aldrich, St. Louis, MO, USA) OPD. The final reaction was stopped with 50 *μ*l of sulfuric acid 2 N and read at 490 nm using a VictorX3 microplate reader (PerkinElmer, USA). Results were expressed as mean ± standard deviation (SD) of the inverse from the last dilution reached before the cut-off. All experiments were done in triplicate.

### 2.6. Lymphoproliferation Assay

Mice were sacrificed thirty days after the last immunization and their spleens were extracted under aseptic conditions to be homogenized in RPMI 1640 (Gibco) medium [25]. Cells were adjusted to a concentration of 4 × 10^6^ viable cells/ml in RPMI medium supplemented (10% heat inactivated fetal bovine serum plus 50 IU/ml penicillin, 50 *μ*g/ml streptomycin, and 0.25 *μ*g/ml amphotericin B) and cultured (100 *μ*l per well) in 96-well microtiter plates (Nunc, Denmark), previously sensitized with 2 or 10 *μ*g/ml recombinant proteins or crude* B. abortus* RB51 proteins (CBPs), respectively, for 72 h at 37°C and 5% CO_2_. CBPs were obtained from 60% methanol inactivated* Brucella* subjected to a treatment with a hypertonic salt solution (1 M NaCl and 0.1 M sodium citrate) for 24 h; then the bacterial suspension was subjected to sonication for 20 minutes and centrifuged to obtain the supernatant [26]. After culturing, splenocytes were pulsed with 0.5 *μ*Ci tritiated thymidine (^3^H-TdR) per well (Amersham, Life Science, London, UK) and 8 h later radioactivity incorporated into the DNA was measured using a scintillation counter (Beckman LS 6500, USA). As a lymphoproliferation positive control, 10 *μ*g/ml of concanavalin A (Promega, Madison, WI, USA) was used, while complete RPMI 1640 was used as a negative control. All experiments were done in triplicate.

### 2.7. Cytokines Levels

The levels of IFN-*γ* and IL-4 secreted were quantified from the supernatant of splenocytes cultures by antigen-capture ELISA. For this, 10^6^ splenocytes/ml were cultured in 24 wells plates (Nunclon, Denmark) previously sensitized with 2 or 10 *μ*g/ml of recombinant proteins for 72 h at 37°C with 5% CO_2_. Aliquots from each well were then taken and IFN-*γ* and IL-4 levels were measured using the Ready-SET-Go! ELISA kit (eBioscience, Thermo Fisher Scientific, MA, USA) following the manufacturer's instructions. Final cytokine concentrations were calculated using standard curves for recombinants IFN-*γ* and IL-4 (eBioscience, Thermo Fisher Scientific, MA, USA). The final reaction was read at 490 nm on a VictorX3 ELISA reader instrument (PerkinElmer, USA). All experiments were done in triplicate.

### 2.8. Protection Experiment

Thirty days after the last immunization, mice were challenged by intraperitoneal injection with* B. abortus* 2308 at a dose of 10^4^ colony-forming units (CFU) per animal. The positive control corresponded to a group of mice immunized with 1 × 10^8^ CFU of the* B. abortus* RB51 vaccine strain. After fifteen days, all mice were euthanized and their spleens removed. Spleens were homogenized in PBS, serially diluted, and cultured in Petri dishes containing agar Columbia supplemented with 5% sheep blood (bioMérieux, Santiago, Chile) for 72 h at 37°C. Finally, the number of CFU per spleen was calculated and reported as units of protection represented by the reduction of mean ± SD of Log_10_ CFU/spleen relative to the PBS group [17].

### 2.9. Statistical Analysis

Antibody production, levels of cytokines, and lymphoproliferative response were analyzed by two-way ANOVA, while the protective response was analyzed with a one-way ANOVA. Multiple comparisons were made using the Tukey test. Data analyses were performed using the GraphPad Prism 5.0 statistical program. A* P* value of 0.05 or less was considered statistically significant.

## 3. Results

### 3.1. Construction of Multivalent DNA Vaccines and Recombinant Products Purification

The multivalent DNA vaccines were successfully constructed cloning the sequences BAB1_0273-*sodC* (768 bp), BAB1_0278-*sodC* (1071 bp), and BAB1_0273-278-*sodC* (1329 bp) into pVAX1 vector (3000 bp) multicloning site, using* Pst*I and* Bam*HI restriction enzymes (see Figure S1 in Supplementary Material available online at https://doi.org/10.1155/2017/6535479). The recombinant vectors pV273*-sod*, pV278*-sod,* and pV273-278*-soC* were used to induce a specific immune response of BALB/c mice against recombinant antigens codified in these multivalent DNA sequences. The recombinant proteins R273S (27.8 kDa), R278S (40 kDa), and R273-278S (49.3 kDa) were expressed in* E. coli* BL21 (DE3) transformed with the plasmids pQ273s, pQ278s, or pQ273-278s (Figure S2A). All recombinant proteins were tagged with 6x His and visualized by western blot (Figure S2B).

### 3.2. Production of Antibodies

**Figure 1:**
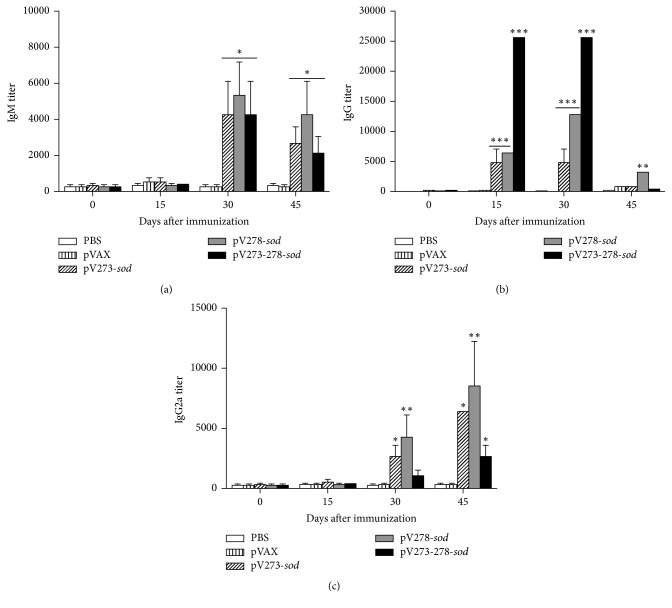
*Peripheral blood antibody production quantified by indirect ELISA*. (a) IgM, (b) IgG, and (c) IgG2a, specific against recombinant proteins. Results are plotted as mean ± standard deviation of the inverse of the last dilution before cutting. ^*∗*^*P* < 0.05, ^*∗∗*^*P* < 0.01, and ^*∗∗∗*^*P* < 0.001 indicate statistically significant values.

Titers of IgM, IgG, IgG1, and IgG2a serum antibodies were measured by ELISA from mice immunized with multivalent DNA vaccines pV273-*sod*, pV278-*sod*, pV273-278-*sod*, pVAX vector or control PBS. Sera of mice 30 days after immunization with different multivalent DNA vaccines achieved a significantly level of anti-recombinant IgM (*P* < 0.05). The levels of specific IgM decreased at day 45 but were still significantly greater than the PBS and pVAX control groups (*P* < 0.05) (Figure 1(a)). Mice immunized with pV273-*sod*, pV278-*sod,* or pV273-278-*sod* had significantly increased titers of the specific IgG at days 15 and 30 after immunization when compared to pVAX and PBS control groups, these levels being higher in the group immunized with the trivalent pV273-278-*sod* vaccine (*P* < 0.001). Significant levels of type-IgG antibodies were observed in sera from immunized animals with pV278-*sod* vaccines at day 45 after immunization (*P* < 0.01), where response on this same day was low and not significant when compared to mice immunized with the other DNA vaccines under study (Figure 1(b)). When evaluating IgG2a levels, it was observed that the bivalent DNA vaccines pV273 and pV278-*sod* produced a significant increase of this antibody at day 30 following vaccination and the peak titers were detected at day 45 after immunization (end of experiment) (*P* < 0.05 and *P* < 0.01, resp.). Trivalent vaccine pV273-278-*sod* produced a significant level of IgG2a only at day 45 after immunization (*P* < 0.05) (Figure 1(c)). None of the studied animals inoculated with different DNA vaccines induced production of specific IgG1 (data not shown).

### 3.3. Lymphoproliferative Response

**Figure 2:**
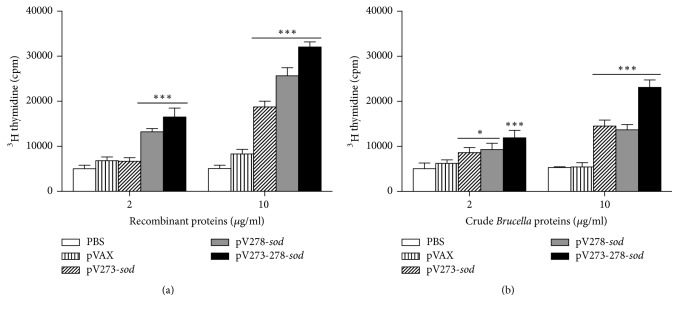
*Lymphoproliferative response*. Splenocytes stimulated in vitro with (a) 2 *μ*g/ml or 10 *μ*g/ml of R273S, R278S, or R273-278S recombinant proteins, respectively, and (b) 2 *μ*g/ml or 10 *μ*g/ml of* B. abortus* 2308 crude* Brucella* proteins. Results are plotted as mean ± standard deviation. ^*∗*^*P* < 0.05 and ^*∗∗∗*^*P* < 0.001 indicate statistically significant values.

Results showed that splenocytes of mice immunized with pV278-*sod* or pV273-278-*sod* vaccines proliferated significantly after being stimulated with 2 *μ*g/ml of their respective recombinant proteins (*P* < 0.001), while pV273*-sod* vaccine did not induce differences compared to the control group (*P* > 0.05) (Figure 2(a)). Splenocytes from mice immunized with pV273-*sod*, pV278-*sod,* or pV273-278-*sod* vaccines had a significant T-cell proliferation response using 10 *μ*g/ml of their respective recombinant proteins (*P* < 0.001) (Figure 2(a)). In vitro stimulation of splenocytes from mice immunized with pV273-*sod* or pV278-*sod* vaccines with 2 *μ*g/ml of crude* Brucella* proteins (CBPs) significantly increased their cellular proliferation (*P* < 0.05). The proliferative response was more pronounced and significant when stimulating with 2 *μ*g/ml of CBPs the splenocytes obtained from the group of animals immunized with the trivalent vaccine pV273-278-*sod* (*P* < 0.001) (Figure 2(a)). On the other hand, when splenocytes from mice immunized with the pV273-*sod*, pV278-*sod,* or pV273-278-*sod* vaccines were stimulated in vitro with 10 *μ*g/ml of CBPs they showed significant proliferation when compared to control groups (*P* < 0.001) (Figure 2(b)). The positive control (10 *μ*g/ml of ConA) showed a higher lymphoproliferative response than the experimental group (data not shown).

### 3.4. Levels of Cytokines

**Figure 3:**
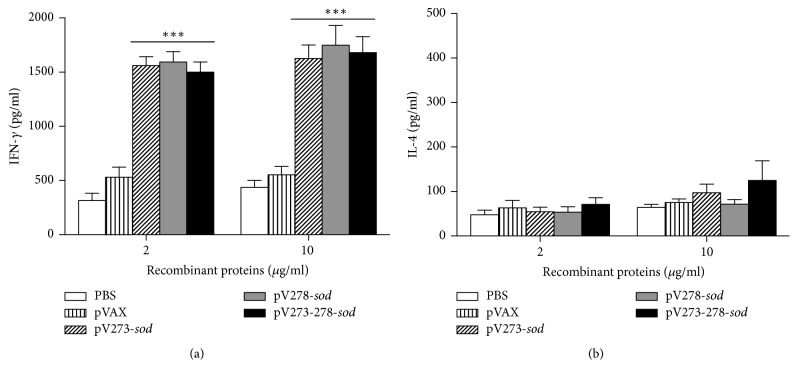
*Cytokine levels quantified by ELISA sandwich*. Production of (a) IFN-*γ* and (b) IL-4, present in the supernatant of splenocytes stimulated in vitro with 2 or 10 *μ*g/ml of R273S, R278S, or R273-278S recombinant proteins for 72 h. Results are plotted as mean ± standard deviation. ^*∗∗∗*^*P* < 0.001 indicates statistically significant values.

The Th1/Th2 response induced in the immunized mice was evaluated in vitro by the production of IFN-*γ* and IL-4 in splenocytes stimulated with the respective recombinant proteins. Splenocytes from mice immunized with multivalent DNA vaccines pV273-*sod*, pV278-*sod,* or pV273-278-*sod* significantly increased IFN-*γ* production after being stimulated with 2 or 10 *μ*g/ml of recombinant proteins, reaching highly significant* P* values when compared to control groups PBS and pVAX (*P* < 0.001) (Figure 3(a)). In vitro stimulation of splenocytes with 2 or 10 *μ*g/ml recombinant proteins did not produce a significant increase in IL-4 production (Figure 3(b)).

### 3.5. Protective Response

The protective response was quantified by the ability of the immunized mice to eliminate the* B. abortus* 2308 pathogenic strain from the spleen. Results showed that immunization with the pV273-*sod* vaccine reduced the CFU by log_10_ 0.4 when compared to the PBS injected group. Meanwhile, immunization with the pV278-*sod* and pV273-278-*sod* vaccines reduced the CFU by log_10_ 0.32 and 0.28, respectively, when compared to the PBS group. These results are not statistically significant (*P* > 0.05) when compared to the PBS control group. The positive control group reduced the CFU by log_10_ 0.94 (*P* < 0.05). Results showed that the three multivalent DNA vaccines conferred lower levels of protection against the pathogenic* B. abortus* 2308 strain (Table 1).

## 4. Discussion

In order to obtain a safer vaccine against brucellosis, immunization with DNA vectors has been implemented due to their ability to induce the generation of a Th1-type immune response, which is protective against* B. abortus* [14, 27, 28]. This response can be induced by immunization, allowing its rapid activation at the time of infection with* Brucella*, where its immunogenic antigens are processed and presented to T cells [29]. DNA immunization using BAB1_0278 ORF [19, 20], immunodominant epitopes of BAB1_0273 ORF [21], and Cu-Zn superoxide dismutase gene* (sodC)* [17] appeared to be highly immunogenic and capable of conferring high levels of protection in mice challenged with pathogenic strain* B. abortus* 2308. Based on this background, we built the multivalent DNA vaccines pV273-*sod*, pV278-*sod,* and pV273-278-*sod* by combining BAB1_0273 or/and BAB1_0278 ORFs to the* B. abortus sodC* gene in order to examine whether these vaccine designs can induce an effective protective immune response against pathogenic strain* B. abortus* 2308 in BALB/c mice.

The immunization with these multivalent DNA vaccines activated humoral and cellular immune responses in mice. Humoral immunity was quantified by the production of serum IgM, IgG, IgG1, and IgG2a antibodies. All multivalent vaccines induced a significant increase of specific IgM and IgG antibodies response that decays over time, the trivalent vaccine pV273-278-*sod* being the one producing the highest titer of specific IgG antibody. Levels of different IgG isotypes showed a predominant production of IgG2a in mice immunized with the three vaccines. IgG2a is produced by plasma cells in the presence of IFN-*γ* [30], a cytokine associated with an efficient protection against intracellular pathogens, such as* Brucella* [31]. This isotype promotes phagocytosis of* Brucella*, accelerating phagosome maturation and activating a wide range of antimicrobial responses in phagocytic cells [14]. This response allows the elimination of phagocytized bacteria followed by the activation of cellular immune responses through the presentation of antigens, costimulatory proteins, and cytokines [28]. Although these antibodies activate several host protective mechanisms, opsonization of* B. abortus* facilitates pathogen entry into the cells, an environment in which it is highly adapted to survive [32, 33].

Immune mechanisms allowing removal of* B. abortus* from infected cells depend on Th1-type cellular immunity [14, 28]. This response was quantified in vitro by the lymphoproliferative response of T cells and IFN-*γ* levels produced by splenocytes stimulated with recombinant antigens. Results demonstrated that the splenocytes derived from mice immunized with any of the vaccines proliferated significantly when stimulated with the respective recombinant protein or CBPs (2 or 10 *μ*g/ml, resp.). Moreover, splenocytes from mice immunized with any of the three DNA vaccines and stimulated with the respective recombinant protein induced IFN-*γ* production, a fundamental cytokine in host resistance against brucellosis [31, 34]. IFN-*γ* produces pleiotropic effects that activate different cells of the innate and adaptive immunity, which altogether greatly reduced* B. abortus* infection [14, 34]. These results demonstrated that in vivo expression of BAB1_0273 and/or BABl_0278 antigens conjugated to the SOD protein polarizes mice immunity towards a Th1-type phenotype, a fundamental response in the host cleansing process of* B. abortus* [14, 28, 34].

Nevertheless, when we evaluated if the Th1-type immune response induced by vaccines protects BALB/c mice challenged with pathogenic* B. abortus* 2308 strain, low protection levels were obtained, although independently BAB1_0278 ORF or* sodC* has provided high levels of protection [17, 18]. These levels of protection showed the ability of* B. abortus* to counteract the development of a protective Th1 immune response by impairing the secretion of IL-12 and inhibiting T-cell activity [35] and demonstrate that the immunogenicity of antigens by themselves is essential to induce an immune response, but it is not a parameter sufficient to achieve an effective protective immunity [36]. Interestingly, if we analyze the protection trials described here, we observed a lower number of CFU in the spleen of the animals immunized with different DNA vaccines or immunized with the RB51 vaccine strain when compared to what was described in previous reports [26]. These results could be expected due to the great variability in the protection index (dependent variable) obtained with different DNA vaccines in BALB/c mice (independent variable), which is not yet completely standardized [37]. However, RB51-immunized mice induced approximately 1–1.5 log CFU difference in the spleen of BABL/c mice [38], something relatively similar to what was observed in this study.

Finally, results indicated that pV273-278-sod trivalent vaccine induced higher or similar immunogenicity than pV273-sod and pV278-sod vaccines, including lymphoproliferative response, IFN-*γ*, and several Ig subclasses' production; nevertheless, paradoxically, this vaccine was the one conferring the lower levels of protection against the* B. abortus* 2308 strain. Therefore, we concluded that immunization with multivalent DNA vaccines encoding GI-3 BAB1_0273 or/and BAB1_0278 ORFs conjugated to the* B. abortus* sodC gene polarizes mice immunity to a Th1-type phenotype, but this immunogenicity was insufficient to induce significant levels of protection in this animal model.

## Supplementary Material

Figure S1. Electrophoresis agarose gel of multivalent DNA vaccines digested with BamHI and PstI restriction enzymes. Lane 1 and 2: DNA size marker (1 kb and 100 bp DNA ladder, respectively); lane 3: pVAX (3000 bp) plus BAB1_0273-sod (768 bp); lane 4: pVAX plus BAB1_0278-sod (1071 bp); and lane 4: pVAX plus BAB1_0273-278-sod (1329 bp). Figure S2. Expression and purification of different recombinant proteins. A) SDS-PAGE analysis. MW, molecular weight (PageRuler Pre-Stained Protein Ladder); lane 1: R273S (27.8 kDa); lane 2: R278S (40 kDa) and lane 3: R273-278S (49.3 kDa). B) Western Blot analysis of recombinant proteins with anti-His-tag monoclonal antibody. Lane 1: R273S; lane 2: R278S, and lane 3: 273-278S.

## Figures and Tables

**Table 1:** Protection conferred by multivalent DNA vaccines in BALB/c mice challenged with the pathogenic *B. abortus* 2308 strain.

Experimental groups (*n* = 5)	Log_10_ CFU of *Brucella abortus* 2308 per spleen (means ± SD)	Log_10_ units of protection^a^
PBS	4.98 ± 0.09	0
pVAX	4.92 ± 0.06	0
pV273-*sod*	4.58 ± 0.07	0.40
pV278-*sod*	4.66 ± 0.18	0.32
pV273-278-*sod*	4.7 ± 0.23	0.28
*B. abortus* strain RB51	4.04 ± 0.25	0.94^*∗*^

^a^Units of protection represent the difference between the mean ± SD of Log_10_ CFU values of the PBS control group and log10 CFU values of the immunized group. All animals were challenged with *B. abortus* 2308 strain and bacterial counts were assessed at day 15. ^*∗*^*P* < 0.05 indicates statistically significant values..
