# Supplementary material for: Multivalent Fusion DNA Vaccine against Brucella abortus
Source: Biomed Res Int. 2017 Sep 7;2017:6535479. doi: 10.1155/2017/6535479 (PMC5610864; doi:10.1155/2017/6535479)
Supplement: Supplementary file 1 — Figure S1. Electrophoresis agarose gel of multivalent DNA vaccines digested with BamHI and PstI restriction enzymes. Lane 1 and 2: DNA size marker (1 kb and 100 bp DNA ladder, respectively); lane 3: pVAX (3000 bp) plus BAB1_0273-sod (768 bp); lane 4: pVAX plus BAB1_0278-sod (1071 bp); and lane 4: pVAX plus BAB1_0273-278-sod (1329 bp). Figure S2. Expression and purification of different recombinant proteins. A) SDS-PAGE analysis. MW, molecular weight (PageRuler Pre-Stained Protein Ladder); lane 1: R273S (27.8 kDa); lane 2: R278S (40 kDa) and lane 3: R273-278S (49.3 kDa). B) Western Blot analysis of recombinant proteins with anti-His-tag monoclonal antibody. Lane 1: R273S; lane 2: R278S, and lane 3: 273-278S. [file 6535479.f1.pdf]

Supplementary Materials

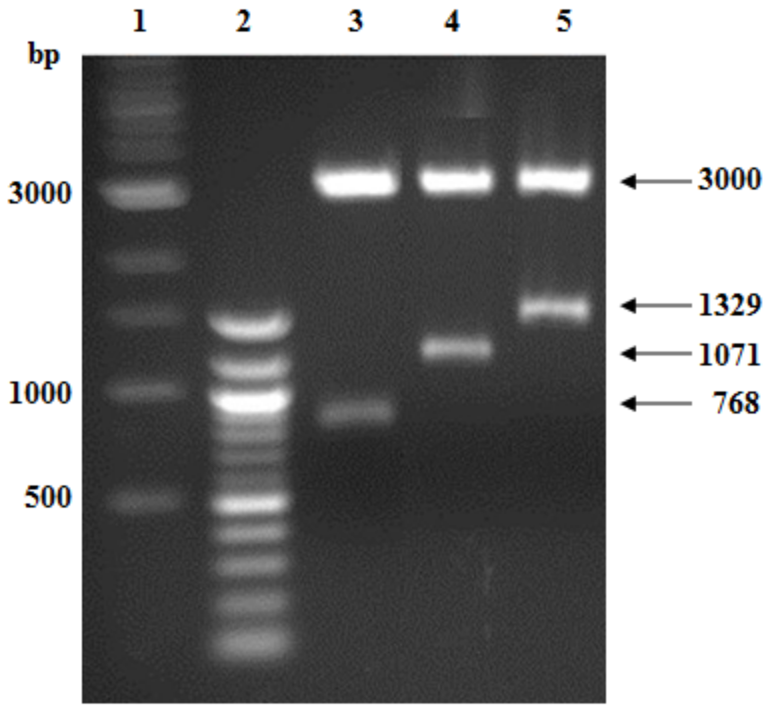

Figure S1. Electrophoresis agarose gel of multivalent DNA vaccines digested with *Bam*HI and *Pst*I restriction enzymes. Lane 1 and 2: DNA size marker (1 kb and 100 bp DNA ladder, respectively); lane 3: pVAX (3000 bp) plus BAB1_0273-*sod* (768 bp); lane 4: pVAX plus BAB1_0278-*sod* (1071 bp); and lane 4: pVAX plus BAB1_0273-278-*sod* (1329 bp).

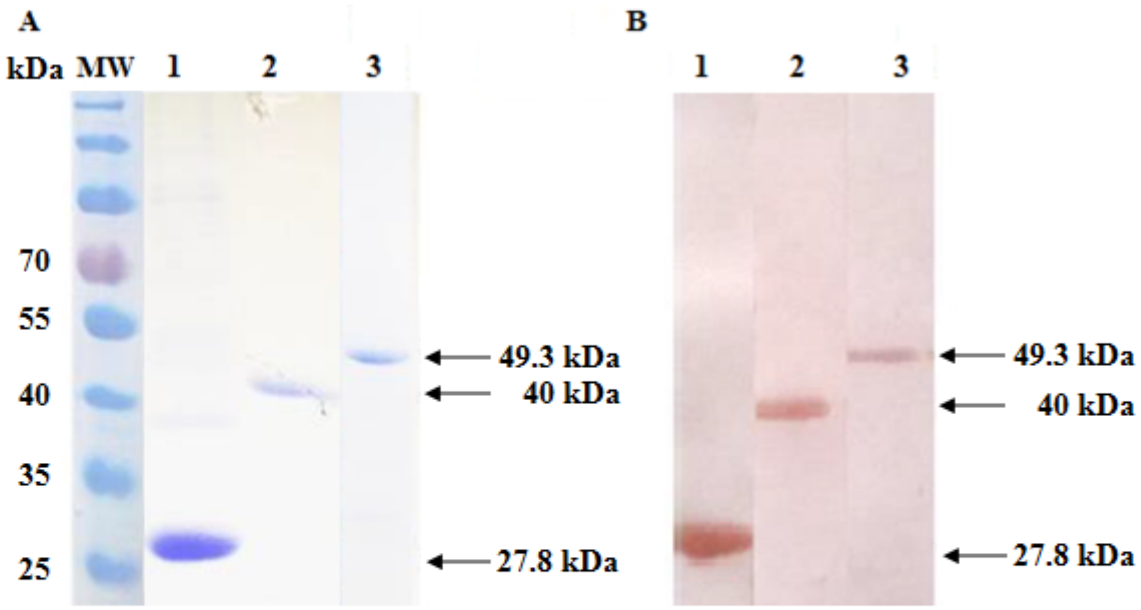

Figure S2. Expression and purification of different recombinant proteins. A) SDS-PAGE analysis. MW, molecular weight (PageRuler Pre-Stained Protein Ladder); lane 1: R273S (27.8 kDa); lane 2: R278S (40 kDa) and lane 3: R273-278S (49.3 kDa). B) Western Blot analysis of recombinant proteins with anti-His-tag monoclonal antibody. Lane 1: R273S; lane 2: R278S, and lane 3: 273-278S
